# How Humans Differ from Other Animals in Their Levels of Morphological Variation

**DOI:** 10.1371/journal.pone.0006876

**Published:** 2009-09-01

**Authors:** Ann E. McKellar, Andrew P. Hendry

**Affiliations:** 1 Department of Biology, Queen's University, Kingston, Canada; 2 Redpath Museum and Department of Biology, McGill University, Montreal, Canada; London School of Economics, United Kingdom

## Abstract

Animal species come in many shapes and sizes, as do the individuals and populations that make up each species. To us, humans might seem to show particularly high levels of morphological variation, but perhaps this perception is simply based on enhanced recognition of individual conspecifics relative to individual heterospecifics. We here more objectively ask how humans compare to other animals in terms of body size variation. We quantitatively compare levels of variation in body length (height) and mass within and among 99 human populations and 848 animal populations (210 species). We find that humans show low levels of within-population body height variation in comparison to body length variation in other animals. Humans do not, however, show distinctive levels of within-population body mass variation, nor of among-population body height or mass variation. These results are consistent with the idea that natural and sexual selection have reduced human height variation within populations, while maintaining it among populations. We therefore hypothesize that humans have evolved on a rugged adaptive landscape with strong selection for body height optima that differ among locations.

## Introduction

Variation is the raw material for evolution, and it is ubiquitous both within and among populations [1]. However, the balance between forces enhancing variation and forces eroding it likely differs among populations and species. Accordingly, the magnitude of morphological variation can differ markedly among species [1]. As humans, how do we compare to other animals in terms of this variation? Taking a subjective look, morphological variation in a crowd of people might seem large compared to the apparent uniformity of an animal group, such as a flock of birds or a shoal of fish. But perhaps this apparent contrast between humans and other animals is simply a matter of our perception – that is, evolution has probably shaped animals to be more discriminating among individual conspecifics than among individual heterospecifics [2], [3]. Alternatively, contemporary human populations might indeed show greater morphological variation than other species. Possible reasons might include relaxed natural selection on some human traits [4] (although perhaps not on others [5]), the great diversity of conditions we can (and do) inhabit, and recurrent migration and gene flow [6] among populations. Or perhaps humans instead show lower levels of variation – a point we will return to later.

Our goal is to quantitatively determine how levels of morphological variation within humans compare to those in other animal species. We use body size as our focal morphological variable because this trait can be logically compared among species, and because body size data are readily available for a wide variety of animal populations, both human and non-human (see Tables S1 and S2). In an effort to obtain unbiased data, we searched the literature for means and variances in body height or body length (these two terms are here used interchangeably, depending on context) and body mass both within and among populations of humans and other animals. From these data, we calculated the coefficient of variation (CV; standard deviation divided by the mean) as a standardized measure of variance among individuals within populations and among population means. In total, our dataset included body size variation from 55 studies (99 populations) of humans and 107 studies (210 species and 848 populations) of other animals (Tables S1 and S2).

## Results and Discussion

**Figure 1:**
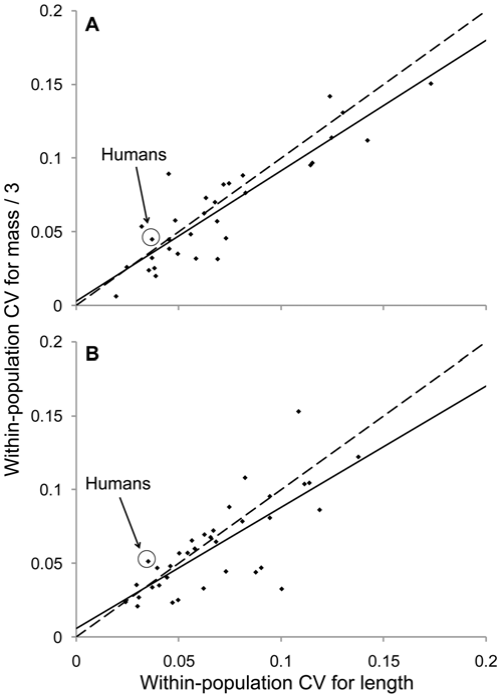
Species-mean CVs for within-population mass (divided by three; see Materials and Methods) versus length. Shown are regression lines (solid), x = y lines (dashed), and data for males (A, R^2^ = 0.81, P<0.001) and females (B, R^2^ = 0.58, P<0.001).

One interesting result was that humans, in comparison to other animals, show a high level of within-population variation in mass considering their within-population variation in height (Figure 1). Specifically, when considering residuals from a regression of within-population CVs for mass on within-population CVs for length, human males and females fell into the 71^st^ and 91^st^ percentiles, respectively, for the entire distribution of animal species.

Why, in comparison to other animals, do humans show high variation in mass relative to height? One contributing factor might be that human height is developmentally determinate, and is therefore relatively stable once an individual reaches maturity. Mass, in contrast, can fluctuate dramatically after maturity based on age, diet, and activity level. In line with this greater environmental (as opposed to genetic) contribution to mass than to length, heritabilities are usually lower for mass than for height in humans [7]–[9]. One important environmental factor contributing particularly to variation in mass might be socioeconomic status. For example, status influences mass differences in both developed and developing countries [10], as well as mass change over time in developed countries [11]. Although socioeconomic status also influences human height, this effect might be more the result of social assortment than variation in nutrition or activity [12], [13]. It is, of course, true that other animals are also influenced by status and nutrition [14]–[16], but perhaps humans have a greater and more consistent availability of the cheap, high energy, processed foods that promote mass gain [17] or greater exposure to societal pressures that contribute to mass loss [18]. Testing these hypotheses for differences between humans and other animals in relative levels of height versus mass variation will require further study.

**Figure 2:**
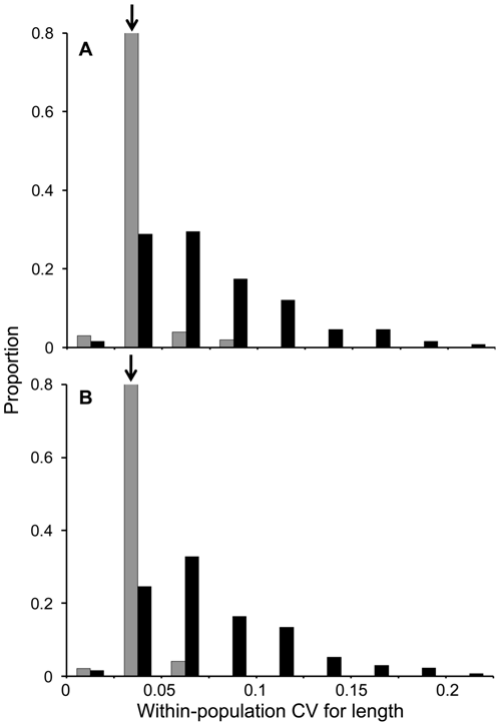
Distributions of coefficients of variation (CV) for within-population body length or height. Shown are species means for animals (black) and population means for humans (grey) for males (A) and females (B). Arrows indicate the locations of CVs for mean human height.

Another interesting result was that humans show low within-population variation in body height in comparison to body length in non-human animals (Figure 2), but the same was not true for human mass relative to animal mass (Figure S1). These differences can be quantified through several different comparisons. First, the mean within-population CVs for male and female human height correspond to the 8^th^ and 4^th^ percentiles, respectively, of the mean within-population CVs for animal length. In contrast, the mean within-population CVs for male and female human mass correspond to the 56^th^ and 60^th^ percentiles, respectively, of the within-population CVs for animal mass. Second, we compared each human population mean individually to the distribution of animal species means – to see whether our results were robust to which particular human population was considered. Here we found that all but 8 of 101 human male samples, and all but 5 of 96 human female samples, had within-population CVs for height that fell below the 25^th^ percentile of the mean within-population CVs for animal length. In contrast, 82 of 98 human male samples and 62 of 90 human female samples fell between the 25^th^ and 65^th^ percentiles of the mean within-population CVs for animal mass. All of the above results are robust to correction for associations between CVs and mean trait sizes (see Methods and Materials).

Why, in comparison to other animals, do humans show low within-population variation in height? The first critical point is that this difference in CVs might reflect differences between humans and other animal species in any of the components of quantitative variation, including additive genetic variance (V_A_), dominance genetic variance (V_D_), epistatic genetic variance (V_I_), maternal effects variance (V_M_), and environmental variance (V_E_) – with the last of these including potential phenotypic plasticity [19]. We are not aware of any studies that directly discriminate among each of these alternatives in a quantitative comparison based on comparable methods applied across many animal species and humans. While acknowledging these possible alternative sources of differences in variation, we here consider the particularly interesting set of hypotheses related to possible differences in V_A_, the currency of adaptation. Thus, differences between species might reflect differences in factors that increase V_A_ (mutation, recombination, gene flow) or decrease V_A_ (stabilizing or directional selection, genetic drift). In view of the wealth of evidence for selection on body size across the animal kingdom [20], we here focus on developing hypotheses related to selection, before later considering some alternatives.

Several possibilities exist for how selection might strongly reduce additive genetic variation for human height. First, some studies have suggested stabilizing natural selection on human height by way of increased health problems in very short and very tall individuals [21], [22]. Second, some studies have suggested directional sexual selection on male human height; taller men often have more sexual partners [21]–[23] and more children [24]. Given that both stabilizing and directional selection should erode genetic variation [25], natural and sexual selection might act together to decrease human height variation. (Note that low genetic variation for height is not incompatible with a significant heritability - if environmental effects are also low.) Perhaps these selective factors are stronger in humans than in other animals – but this has not been studied.

**Figure 3:**
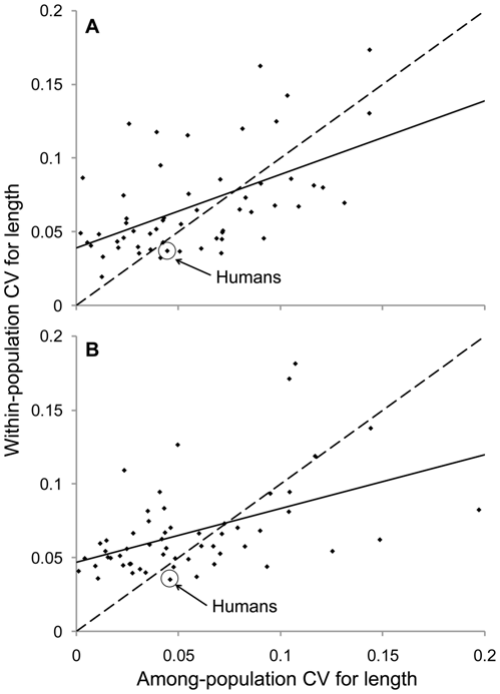
Species-mean CVs for among-population versus within-population body length or height. Shown are regression lines (solid), x = y lines (dashed), and data for males (A, R^2^ = 0.29, P = 0.001) and females (B, R^2^ = 0.23, P<0.001).

Our analyses of among-population variation help to refine the above hypothesis that selection might reduce height variation in humans relative to other animals. In particular, humans show levels of among-population variation in height that are similar to that seen in other animals (Figure S2). Specifically, the mean among-population CVs for male and female human height correspond to the 47^th^ and 51^st^ percentiles, respectively, of mean among-population CVs for animal length. Illustrated another way, humans show relatively low levels of within-population variation in height given their among-population variation in height (Figure 3). Specifically, when considering residuals from a regression of within-population CVs for length on among-population CVs for length, human males and females fall into the 20^th^ and 9^th^ percentiles, respectively.

We hypothesize that this pattern of unremarkable among-population variation in human height, coupled with relatively low within-population variation in human height, is consistent with evolution in response to strong selection for optima that differ among geographic locations. In the lexicon of evolutionary biology [26], the hypothesis is that humans have evolved on a rugged adaptive landscape characterized by sharp fitness peaks that correspond to locally-optimal body sizes that differ among locations. This idea is consistent with several previous arguments for local adaptation in human height. For example, human height increases with increasing latitude [27] (as was also the case in our data set, Figure S3), and with decreasing mean annual temperature [28]. Humans thus follow Bergmann's rule, perhaps because larger bodies are more resistant to heat loss in cold climates – or for other reasons [29]. As another example, the short stature of human pygmies is thought to have evolved via strong selection for small body sizes [30] or life-history trade-offs [31] that characterize their particular tropical forest environments. Our study complements these previous adaptive interpretations by revealing that height variation is low within populations. In short, we hereby add the “rugged” aspect to the existing idea of adaptive peaks that differ among locations.

Several potential complications and alternatives to the role of selection need to be discussed. First, for local adaptation to be substantial, gene flow has to be somewhat limited among populations [32]. This does seem to be the case for humans, at least historically, given the evidence for broad-scale regional clustering of neutral genetic variation [33]–[35]. If populations can diverge appreciably in these neutral genetic markers, then they should be able to diverge easily in response to different selection pressures. Moreover, gene flow might be reduced for genes specifically influencing height because humans often show height-assortative mating [36], [37]. Second, genetic drift is an unlikely explanation for variation in human height among populations because correlations with likely selective factors (e.g., temperature) then would not be so strong and repeatable. Third, plasticity due to geographical differences in childhood nutrition or other environmental factors could account for high variation among, relative to within, populations. Fourth, within-population CVs for human height might be low due to reduced V_E_ rather than reduced V_A_, for instance due to niche construction leading to reduced environmental variance [38]. However, arguing against these latter two possibilities, human mass, which is even more plastic than human height (see above) and is likely influenced by similar environmental factors as is human height, does not show reduced within-population variation relative to among-population variation in comparison to other animals (Figure S4).

In conclusion, we advance the hypothesis that humans have evolved on a rugged adaptive landscape, at least for body height. It would be interesting to see if this hypothesis is supported by analyses of variation in other traits that are shared between humans and other animals. In addition, comparing humans specifically to closely related animal species (i.e., other primates) might give some clue as to whether these forces are specific to humans within the primate order. In any case, we suggest that the adaptive landscape metaphor might provide a useful framework for advancing our understanding of diversification in humans.

## Materials and Methods

We searched the literature for studies reporting means and variation in body size for at least one population of a species. Key words for searches included “body size” and “variation.” Citations from the resulting sources were also examined; for humans, many additional sources were taken from Katzmarzyk and Leonard [28]. Of these studies, we further consider only those that examined wild populations (for non-human animals) and adult individuals (as defined in each study, or 18+ years for humans). If more than one study examined the same population, only the most recent study was used. In total, our dataset (Tables S1 and S2) comprised of 55 studies (99 populations) of humans and 107 studies and 210 species (848 populations) of other animals. This included studies from a variety of animal taxa (10 amphibian, 15 bird, 3 fish, 54 invertebrate, 95 mammal, and 33 reptile) and different types of human populations (e.g., 29 indigenous/aboriginal, 40 Least Developed (http://www.un.org/special-rep/ohrlls/ldc/list.htm)). Raw data is available from the authors upon request. Due to the large size of our animal dataset, and the great diversity of species and populations from across the whole animal phylogeny, we did not apply phylogenetic-based analyses (for a simpler alternative analysis see below).

For each sample, we calculated the within-population coefficient of variation (CV) for body length (height) or mass and then averaged these within-population CVs across the sampled populations. This procedure yielded mean within-population CVs for each species. We calculated among-population CVs by using the mean body size measures for each population. We then evaluated in what percentile human means lie within the overall distribution of animal means. This was done both for distributions of mean values (i.e., Figures 2, S1 and S2) as well as for residuals of regression plots (i.e., Figures 1, 3, and S4). When comparing the relative association between body length and mass CVs among species (i.e., Figure 1), CVs for mass were divided by three so as to be directly comparable in dimensionality to CVs for length [39].

We found no association between CVs and sample sizes either within or among populations for length or mass (results not shown), suggesting that variation in sample size did not influence our results. In contrast, we did find a negative association between trait size (e.g., mean body length) and trait CV (see Houle [40]) within populations for male length (r = −0.35, P<0.01), female length (r = −0.26, P<0.01), male mass (r = −0.27, P<0.01), and female mass (r = −0.24, P<0.01), and among populations for female length (r = −0.063, P = 0.031) but not male length (r = 0.0052, P = 0.56). However, restricting our analysis to animal species with body sizes within the range of human body size did not influence our conclusions that (1) humans have low levels of within-population variation in height (6^th^ percentile for males and 0^th^ percentile for females), but (2) not within-population variation in mass (65^th^ percentile for males and 42^nd^ percentile for females) or (3) among-population variation in height (45^th^ percentile for males and 71^st^ percentile for females).

To assess the generality of our results, we performed the above analyses with various subsets of the data. Our main conclusions, as described above, did not change in any case. We therefore only here list these additional analyses without providing the details. First, the authors of a given study typically defined each “population” as such, or these designations were implicitly obvious. In a few studies, however, the specific populations were less clear (e.g., museum collections over broad regions) – but our conclusions were the same when excluding these more ambiguous cases. Second, conclusions were the same when including or excluding animal species in which tail length was included in body length measurements. Third, conclusions were the same when considering (1) only human studies published before or after 1974 (the median study date, see Table S2), (2) human studies of only indigenous/aboriginal populations (as defined in each study) or only non-indigenous/aboriginal populations, and (3) human studies from only Least Developed Countries (http://www.un.org/special-rep/ohrlls/ldc/list.htm) or only non-Least Developed Countries. Fourth, conclusions were the same when humans were compared specifically to different taxomonic groups (Table S3), although the distinctiveness of within-population CVs for male (but not female) height was less strong (18^th^ percentile) when humans were compared only to other mammals. Overall, then, our conclusions are robust to the inclusion or exclusion of particular human populations or animal species.

## Supporting Information

Table S1 List of all studies, species, and taxa (amphibian, bird, fish, invertebrate, mammal, or reptile) used to obtain coefficients of variation (CV) for male and/or female length and/or mass for animal populations.
(0.25 MB DOC)

Table S2 List of all studies used to obtain CVs for male and/or female height and/or mass for human populations. Also included is the country of origin, name of specific population or survey title, year of sampling (if provided), indigenous/aboriginal status (as defined in each study), and development status (http://www.un.org/special-rep/ohrlls/ldc/list.htm).
(0.11 MB DOC)

Table S3 Percentiles for mean within- and among-population male and female human height and mass in relation to species-mean amphibian, invertebrate, mammal, and reptile length and mass distributions. Percentiles are not shown for taxa distributions with n<5 animal species.
(0.03 MB DOC)

Figure S1 Distributions of coefficients of variation (CV) for within-population body mass. Shown are species means for animals (black) and population means for humans (grey) for males (A) and females (B). Arrows indicate the locations of CVs for mean human mass.
(1.80 MB TIF)

Figure S2 Distributions of CVs for among-population body length or height. Shown are data for males (A) and females (B). Arrows indicate the locations of CVs for mean human height.
(1.72 MB TIF)

Figure S3 Bergmann's rule in humans. Mean male height (A, R^2^ = 0.126, P<0.001), female height (B, R^2^ = 0.097, P = 0.002), male mass (C, R^2^ = 0.183, P<0.001), and female mass (D, R^2^ = 0.155, P<0.001) all increase significantly with absolute latitude. Latitude of each population was approximated using the geographic centre of the country from which the population was sampled. Coordinates were obtained from the CIA World Factbook (https://www.cia.gov/library/publications/the-world-factbook/fields/2011.html).
(1.06 MB TIF)

Figure S4 Species-mean CVs for among- versus within-population body mass. Shown are regression lines (solid), x = y lines (dashed), and data for males (A, R^2^ = 0.26, P<0.001) and females (B, R^2^ = 0.37, P<0.001).
(1.72 MB DOC)
