# Supplementary material for: How Humans Differ from Other Animals in Their Levels of Morphological Variation
Source: PLoS One. 2009 Sep 1;4(9):e6876. doi: 10.1371/journal.pone.0006876 (PMC2730817; doi:10.1371/journal.pone.0006876)
Supplement: Table S1 — List of all studies, species, and taxa (amphibian, bird, fish, invertebrate, mammal, or reptile) used to obtain coefficients of variation (CV) for male and/or female length and/or mass for animal populations. (0.25 MB DOC) [file pone.0006876.s001.doc]

**Table S1. List of all studies, species, and taxa (amphibian, bird, fish, invertebrate, mammal, or reptile)** used to obtain coefficients of variation (CV) for male and/or female length and/or mass for animal populations.

| Species | Taxon | Trait | Sex | Reference |
| --- | --- | --- | --- | --- |
| *Agriphila plumbifimbriella* | Invertebrate | mass | male, female | Marshall, *Am. Midl. Nat.* 119, 412-419 (1988). |
| *Alces alces* | Mammal | mass | male, female | Sand *et al.*, *Oecologia* 102, 433-442 (1995). |
| *Alouatta caraya* | Mammal | mass | male, female | Rumiz, *Am. J. Primatol.* 21, 279-294 (1990). |
| *Alouatta palliata* | Mammal | length, mass | male, female | Glander *et al.*, *Folia Primatol.* 57, 70-81 (1991). |
|  |  |  |  | Thorington, *Vertebrate ecology in the northern neotropics* (1979) p. 97-106. |
| *Alouatta seniculus* | Mammal | length, mass | male, female | Thorington, *Vertebrate ecology in the northern neotropics* (1979) p. 97-106. |
|  |  |  |  | Braza *et al.*, *Mammalia* 47, 205-214 (1983). |
| *Amegilla sapiens* | Invertebrate | mass | female | Stone, *J. Comp. Physiol. B* 163, 317 (1993). |
| *Anolis sagrei* | Amphibian | length | male | Kolbe *et al.*, *Mol. Ecol.* 16, 1579-1591 (2007). |
| *Anser albifrons* | Bird | mass | male, female | Ely *et al.*, *Bird Study* 52, 104-119 (2005). |
| *Anser caerulescens atlanticus* | Bird | mass | male, female | Reed, Plante, *J. Wildl. Manage.* 61, 413-419 (1997). |
| *Arctopsyche grandis* | Invertebrate | length | male, female | Jannot, Kerans, *Can. J. Zool.* 81, 1956-1964 (2003). |
| *Arctopsyche irrorata* | Invertebrate | length | male, female | Jannot, Kerans, *Can. J. Zool.* 81, 1956-1964 (2003). |
| *Arizona elegans* | Reptile | length | female | Aldridge, *Herpetologica* 35, 256-261 (1979). |
| *Ateles geoffroyi* | Mammal | length, mass | male, female | Glander *et al.*, *Folia Primatol.* 57, 70-81 (1991). |
| *Avahi laniger* | Mammal | length, mass | male, female | Glander *et al.*, *J. Hum. Evol.* 22, 1-17 (1992). |
| *Bothrops mattogrossensis* | Reptile | length | male, female | Monteiro *et al.*, *J. Herpetol.* 40, 408-413 (2006). |
| *Brachyteles arachnoides* | Mammal | length, mass | male, female | Lemos de Sá, Glander, *Am. J. Primatol.* 29, 145-153 (1993). |
| *Bufo viridis* | Amphibian | length, mass | male | Castellano, Giacoma, *J. Herpetol.* 32, 540-550 (1998). |
| *Callosobruchus chinensis* | Invertebrate | mass | male, female | Colgoni, Vamosi, *Entomol. Sci.* 9, 171-179 (2006). |
| *Callosobruchus maculatus* | Invertebrate | mass | male, female | Colgoni, Vamosi, *Entomol. Sci.* 9, 171-179 (2006). |
| *Caretta caretta* | Reptile | length | female | van Buskirk, Crowder, *Copeia* 1994, 66-81 (1994). |
| *Cebus capucinus* | Mammal | length, mass | male, female | Glander *et al.*, *Folia Primatol.* 57, 70-81 (1991). |
| *Ceratopsyche alternans* | Invertebrate | length | male, female | Jannot, Kerans, *Can. J. Zool.* 81, 1956-1964 (2003). |
| *Ceratopsyche cockerelli* | Invertebrate | length | male, female | Jannot, Kerans, *Can. J. Zool.* 81, 1956-1964 (2003). |
| *Ceratopsyche morosa* | Invertebrate | length | male, female | Jannot, Kerans, *Can. J. Zool.* 81, 1956-1964 (2003). |
| *Ceratopsyche oslari* | Invertebrate | length | male, female | Jannot, Kerans, *Can. J. Zool.* 81, 1956-1964 (2003). |
| *Ceratopsyche slossonae* | Invertebrate | length | male, female | Jannot, Kerans, *Can. J. Zool.* 81, 1956-1964 (2003). |
| *Ceratopsyche sparna* | Invertebrate | length | male, female | Jannot, Kerans, *Can. J. Zool.* 81, 1956-1964 (2003). |
| *Cercopithecus aethiops* | Mammal | mass | male, female | Turner *et al.*, *Folia Primatol.* 63, 177-179 (1994). |
| *Chelonia mydas* | Reptile | length | female | van Buskirk, Crowder, *Copeia* 1994, 66-81 (1994). |
| *Cheumatopsyche aphanta* | Invertebrate | length | male, female | Jannot, Kerans, *Can. J. Zool.* 81, 1956-1964 (2003). |
| *Cheumatopsyche gracilis* | Invertebrate | length | male, female | Jannot, Kerans, *Can. J. Zool.* 81, 1956-1964 (2003). |
| *Cheumatopsyche harwoodi* | Invertebrate | length | male, female | Jannot, Kerans, *Can. J. Zool.* 81, 1956-1964 (2003). |
| *Cheumatopsyche minuscula* | Invertebrate | length | male, female | Jannot, Kerans, *Can. J. Zool.* 81, 1956-1964 (2003). |
| *Cheumatopsyche pettiti* | Invertebrate | length | male, female | Jannot, Kerans, *Can. J. Zool.* 81, 1956-1964 (2003). |
| *Cheumatopsyche speciosa* | Invertebrate | length | male, female | Jannot, Kerans, *Can. J. Zool.* 81, 1956-1964 (2003). |
| *Chimarra aterrima* | Invertebrate | length | male, female | Jannot, Kerans, *Can. J. Zool.* 81, 1956-1964 (2003). |
| *Chimarra texana* | Invertebrate | length | male, female | Jannot, Kerans, *Can. J. Zool.* 81, 1956-1964 (2003). |
| *Chrysemys picta bellii* | Reptile | length, mass | male, female | Rowe, *Am. Midl. Nat.* 138, 174-188 (1997). |
| *Cinnycerthia peruana* | Bird | mass | male, female | Brumfield, Remsen, *Wilson Bull.* 108, 205-227 (1996). |
| *Clethrionomys californicus obscurus* | Mammal | length | male, female | Heske, Ostfeld, *J. Mammal.* 71, 510-519 (1990). |
| *Clethrionomys gapperi* | Mammal | length | male, female | Heske, Ostfeld, *J. Mammal.* 71, 510-519 (1990). |
| *Clethrionomys rutilus* | Mammal | length | male, female | Heske, Ostfeld, *J. Mammal.* 71, 510-519 (1990). |
| *Cnemidophorus cryptus* | Reptile | length, mass | female | Vitt *et al.*, *Copeia* 1997, 745-757 (1997). |
| *Cnemidophorus lemniscatus* | Reptile | length, mass | male, female | Vitt *et al.*, *Copeia* 1997, 745-757 (1997). |
| *Coccinella septempunctata* | Invertebrate | length | male, female | Zhou *et al.*, *Entomol. Exp. Appl.* 75, 99-107 (1995). |
| *Corvus corone* | Bird | mass | male, female | Acquarone *et al.*, *Folia Zool.* 53, 379-384 (2004). |
| *Crotalus viridis* | Reptile | length, mass | male, female | Diller, Wallace, *Herpetologica* 40, 182-193 (1984). |
|  |  |  |  | Diller, Wallace, *Herpetologica* 40, 182-193 (1984). |
|  |  |  |  | Graves, Duvall, *J. Herpetol.* 24, 351-356 (1990). |
|  |  |  |  | Gannon, Secoy, *J. Herpetol.* 18, 13-19 (1984). |
|  |  |  |  | Fitch, *Trans. Kansas Acad. Sci.* 101, 101-113 (1998). |
|  |  |  |  | Ashton, Patton, *Copeia* 2001, 229-234 (2001). |
|  |  |  |  | Aldridge, *Herpetologica* 35, 256-261 (1979). |
| *Ctenomys argentinus* | Mammal | length, mass | male, female | Medina *et al.*, *J. Biogeography* 34, 1439-1454 (2007). |
|  |  |  |  | Medina *et al.*, *J. Biogeography* 34, 1439-1454 (2007). |
| *Ctenomys azarae* | Mammal | length, mass | female | Medina *et al.*, *J. Biogeography* 34, 1439-1454 (2007). |
| *Ctenomys bergi* | Mammal | length, mass | male, female | Medina *et al.*, *J. Biogeography* 34, 1439-1454 (2007). |
| *Ctenomys conoveri* | Mammal | length, mass | male | Medina *et al.*, *J. Biogeography* 34, 1439-1454 (2007). |
| *Ctenomys dorbignyi* | Mammal | length, mass | male, female | Medina *et al.*, *J. Biogeography* 34, 1439-1454 (2007). |
| *Ctenomys fodax* | Mammal | length | male | Medina *et al.*, *J. Biogeography* 34, 1439-1454 (2007). |
| *Ctenomys latro* | Mammal | length, mass | male, female | Medina *et al.*, *J. Biogeography* 34, 1439-1454 (2007). |
| *Ctenomys leucodon* | Mammal | length | male | Medina *et al.*, *J. Biogeography* 34, 1439-1454 (2007). |
| *Ctenomys mendocinus* | Mammal | length, mass | male, female | Medina *et al.*, *J. Biogeography* 34, 1439-1454 (2007). |
| *Ctenomys opimus* | Mammal | length, mass | female | Medina *et al.*, *J. Biogeography* 34, 1439-1454 (2007). |
| *Ctenomys perrensi complex* | Mammal | length, mass | male, female | Medina *et al.*, *J. Biogeography* 34, 1439-1454 (2007). |
| *Ctenomys pilarensis* | Mammal | length, mass | male, female | Medina *et al.*, *J. Biogeography* 34, 1439-1454 (2007). |
| *Ctenomys pundti* | Mammal | mass | male | Medina *et al.*, *J. Biogeography* 34, 1439-1454 (2007). |
| *Ctenomys roigi* | Mammal | length, mass | male, female | Medina *et al.*, *J. Biogeography* 34, 1439-1454 (2007). |
| *Ctenomys rosendopascuali* | Mammal | length, mass | male, female | Medina *et al.*, *J. Biogeography* 34, 1439-1454 (2007). |
| *Ctenomys talarum* | Mammal | length, mass | male, female | Medina *et al.*, *J. Biogeography* 34, 1439-1454 (2007). |
|  |  |  |  | Zenuto, *J. Nat. Hist.* 33, 305-314 (1999). |
| *Ctenomys tuconax* | Mammal | length, mass | male, female | Medina *et al.*, *J. Biogeography* 34, 1439-1454 (2007). |
| *Ctenomys tucumanus* | Mammal | length, mass | male, female | Medina *et al.*, *J. Biogeography* 34, 1439-1454 (2007). |
| *Ctenomys viperinus* | Mammal | length, mass | male, female | Medina *et al.*, *J. Biogeography* 34, 1439-1454 (2007). |
| *Dermochelys coriacea* | Reptile | length | male, female | Witt *et al.*, *Mar. Ecol. Prog. Ser.* 337, 231-243 (2007). |
| *Desmognathus fuscus fuscus* | Amphibian | length | male, female | Davic, *Copeia* 1983, 1101-1104 (1983). |
| *Desmognathus monticola* | Amphibian | length | male, female | Bruce, Hairston, *J. Herpetol.* 24, 124-134 (1990). |
| *Desmognathus ochrophaeus* | Amphibian | length | female | Tilley, *Ecology* 54, 3-17 (1973). |
| *Dichroplus pratensis* | Invertebrate | length | male, female | Bidau, Martí, *Ann. Entomol. Soc. Am.* 100 , 850-860 (2007). |
| *Dichroplus vittatus* | Invertebrate | length | male, female | Bidau, Martí, *Ann. Entomol. Soc. Am.* 100 , 850-860 (2007). |
| *Diplectrona modesta* | Invertebrate | length | male, female | Jannot, Kerans, *Can. J. Zool.* 81, 1956-1964 (2003). |
| *Dipodomys merriami* | Mammal | mass | male, female | Koontz *et al.*, *J. Arid Environ.* 49, 581-591 (2001). |
| *Dipodomys simulans* | Mammal | length | male, female | Sullivan, Best, *J. Mammal.* 78, 798-810 (1997). |
| *Dolophilodes aequalis* | Invertebrate | length | male, female | Jannot, Kerans, *Can. J. Zool.* 81, 1956-1964 (2003). |
| *Dolophilodes distinctus* | Invertebrate | length | male, female | Jannot, Kerans, *Can. J. Zool.* 81, 1956-1964 (2003). |
| *Elaphe quatuorlineata* | Reptile | length | male, female | Filippi *et al.*, *Copeia* 2005, 517-525 (2005). |
| *Eleutherodactylus glaphycompus* | Amphibian | length, mass | male, female | Hedges, *J. Herpetol.* 25, 10-17 (1991). |
| *Embernagra platensis complex* | Bird | mass | male, female | Hayes, *Ardeola* 50, 223-235 (2003). |
| *Emys orbicularis* | Reptile | length | male, female | Zuffi, *J. Zool. (London)* 247, 139-143 (1999). |
| *Eretmochelys imbricata* | Reptile | length | female | van Buskirk, Crowder, *Copeia* 1994, 66-81 (1994). |
| *Eulemur fulvus rufus* | Mammal | length, mass | male, female | Glander *et al.*, *J. Hum. Evol.* 22, 1-17 (1992). |
| *Eulemur rubriventer* | Mammal | length, mass | male, female | Glander *et al.*, *J. Hum. Evol.* 22, 1-17 (1992). |
| *Falco sparverius* | Bird | mass | male, female | Layne, Smith, *J. Field Ornithol.* 63, 256-263 (1992) |
| *Hapalemur aureus* | Mammal | length, mass | male | Glander *et al.*, *J. Hum. Evol.* 22, 1-17 (1992). |
| *Henosepilachna niponica* | Invertebrate | length | male, female | Ohgushi, *Res. Pop. Ecol.* 29, 147-154 (1987). |
| *Hydropsyche betteni* | Invertebrate | length | male, female | Jannot, Kerans, *Can. J. Zool.* 81, 1956-1964 (2003). |
| *Hydropsyche confusa* | Invertebrate | length | male, female | Jannot, Kerans, *Can. J. Zool.* 81, 1956-1964 (2003). |
| *Hydropsyche elissoma* | Invertebrate | length | male, female | Jannot, Kerans, *Can. J. Zool.* 81, 1956-1964 (2003). |
| *Hydropsyche hageni* | Invertebrate | length | male, female | Jannot, Kerans, *Can. J. Zool.* 81, 1956-1964 (2003). |
| *Hydropsyche occidentalis* | Invertebrate | length | male, female | Jannot, Kerans, *Can. J. Zool.* 81, 1956-1964 (2003). |
| *Hydropsyche scalaris* | Invertebrate | length | male, female | Jannot, Kerans, *Can. J. Zool.* 81, 1956-1964 (2003). |
| *Hyla intermedia* | Amphibian | length | male | Rosso *et al.*, *Evol. Ecol.* 18, 303-321 (2004). |
| *Hynobius naevius* | Amphibian | length | male | Tominaga *et al.*, *Zool. Sci.* 22, 1229-1244 (2005). |
| *Isoodon obesulus* | Mammal | mass | male, female | Cooper, *Aust. J. Zool.* 46, 145-152 (1998). |
| *Larus argentatus* | Bird | mass | male, female | Monaghan *et al.*, *Ibis* 125, 412-417 (1983). |
| *Larus audouinii* | Bird | mass | male, female | Ruiz *et al.*, *Ibis* 140, 431-438 (1998). |
|  |  |  |  | Genovart *et al.*, *Ibis* 145, 448-456 (2003). |
| *Lepidochelys kempii* | Reptile | length | female | van Buskirk, Crowder, *Copeia* 1994, 66-81 (1994). |
| *Lepidochelys olivacea* | Reptile | length | female | van Buskirk, Crowder, *Copeia* 1994, 66-81 (1994). |
| *Lepilemur ruficaudatus* | Mammal | mass | male, female | Schmid, Ganzhorn, *Am. J. Primatol.* 38, 169-174 (1996). |
| *Leptonema albovirens* | Invertebrate | length | male, female | Jannot, Kerans, *Can. J. Zool.* 81, 1956-1964 (2003). |
| *Limnodynastes peronii* | Reptile | length | male, female | Schäuble, *Biol. J. Linnean Soc.* 82, 39-56 (2004). |
| *Limnodynastes tasmaniensis* | Reptile | length | male, female | Schäuble, *Biol. J. Linnean Soc.* 82, 39-56 (2004). |
| *Liophis miliaris* | Reptile | length | male, female | Pizzatto, Marques, *Amphib.-Reptilia* 27, 37-46 (2006). |
| *Lophocebus albigena* | Mammal | mass | male | Olupot, 2000 *Conserv. Biol.* 14, 833-843 (2000). |
| *Lype diversa* | Invertebrate | length | male, female | Jannot, Kerans, *Can. J. Zool.* 81, 1956-1964 (2003). |
| *Macaca fuscata* | Mammal | mass | male, female | Paterson, *Int. J. Primatol.* 17, 585-611 (1996). |
| *Macaca mulatta* | Mammal | length, mass | male, female | Clarke, O’Neil, *Am. J. Primatol.* 47, 335-346 (1999). |
| *Macaca sinica* | Mammal | length, mass | male, female | Cheverud *et al.*, *J. Hum. Evol.* 23, 51-77 (1992). |
| *Macrostemum carolina* | Invertebrate | length | male, female | Jannot, Kerans, *Can. J. Zool.* 81, 1956-1964 (2003). |
| *Macrostemum zebratum* | Invertebrate | length | male, female | Jannot, Kerans, *Can. J. Zool.* 81, 1956-1964 (2003). |
| *Mandrillus sphinx* | Mammal | mass | male, female | Popp, *Primates* 24, 198-210 (1983). |
| *Mastomys huberti* | Mammal | length, mass | male, female | Ganem *et al.*, *Experientia* 51, 402-410 (1995). |
| *Melospiza melodia* | Bird | mass | male, female | Smith, *Zool. J. Linnean Soc.* 122, 427-454 (1998). |
| *Metriaclima zebra* | Fish | length | male | Streelman *et al.*, *Freshw. Biol.* 52, 525-538 (2007). |
| *Microcebus murinus* | Mammal | mass | male, female | Lahann *et al.*, *Int. J. Primatol.* 27, 983-999(2006). |
| *Microcebus rufus* | Mammal | mass | male, female | Atsalis, *Feeding Ecology and Aspects of Life History in* Microcebus rufus *(Family Cheirogaleidae, Order Primates)*, PhD (1997). |
| *Microtus breweri* | Mammal | length | male, female | Heske, Ostfeld, *J. Mammal.* 71, 510-519 (1990). |
| *Microtus californicus aestuarinus* | Mammal | length | male, female | Heske, Ostfeld, *J. Mammal.* 71, 510-519 (1990). |
| *Microtus longicaudus* | Mammal | length | male, female | Heske, Ostfeld, *J. Mammal.* 71, 510-519 (1990). |
| *Microtus mexicanus* | Mammal | length | male, female | Heske, Ostfeld, *J. Mammal.* 71, 510-519 (1990). |
| *Microtus miurus murei* | Mammal | length | male, female | Heske, Ostfeld, *J. Mammal.* 71, 510-519 (1990). |
| *Microtus montanus nanus* | Mammal | length | male, female | Heske, Ostfeld, *J. Mammal.* 71, 510-519 (1990). |
| *Microtus ochrogaster haydent* | Mammal | length | male, female | Heske, Ostfeld, *J. Mammal.* 71, 510-519 (1990). |
| *Microtus oeconomus* | Mammal | length | male, female | Heske, Ostfeld, *J. Mammal.* 71, 510-519 (1990). |
| *Microtus pennsylvanicus* | Mammal | length, mass | male, female | Heske, Ostfeld, *J. Mammal.* 71, 510-519 (1990). |
|  |  |  |  | Hansen, Boostra, *Oikos* 89, 81-94 (2000). |
| *Microtus pinetorum nemoralis* | Mammal | length | male, female | Heske, Ostfeld, *J. Mammal.* 71, 510-519 (1990). |
| *Microtus townsendii* | Mammal | length | male, female | Heske, Ostfeld, *J. Mammal.* 71, 510-519 (1990). |
| *Microtus xanthognathus* | Mammal | length | male, female | Heske, Ostfeld, *J. Mammal.* 71, 510-519 (1990). |
| *Mogera imaizumii* | Mammal | mass | male, female | Hashimoto, Abe, *Mamm. Study* 26, 35-44 (2001). |
| *Mogera tokudae* | Mammal | mass | male, female | Hashimoto, Abe, *Mamm. Study* 26, 35-44 (2001). |
| *Mustela erminea* | Mammal | mass | male, female | Erlinge, *Holarct. Ecol.* 10, 33-39 (1987). |
|  |  |  |  | King, Moody, *N. Z. J. Zool.* 9, 49-144 (1982). |
| *Natatar depressa* | Reptile | length | female | van Buskirk, Crowder, *Copeia* 1994, 66-81 (1994). |
| *Neocalanus cristatus* | Invertebrate | length | male | Tsuda *et al.*, *J. Oceanogr.* 57, 341-352 (2001). |
| *Neocalanus flemingeri* | Invertebrate | length | male, female | Tsuda *et al.*, *J. Oceanogr.* 57, 341-352 (2001). |
|  |  |  |  | Kobari, Ikeda, *Mar. Ecol. Prog. Ser.* 209, 243-255 (2001). |
| *Neocalanus plumchrus* | Invertebrate | length | male, female | Tsuda *et al.*, *J. Oceanogr.* 57, 341-352 (2001). |
| *Nerodia sipedon* | Reptile | length | male, female | King, *Herpetologica* 45, 84-88 (1989). |
| *Neureclipsis bimaculata* | Invertebrate | length | male, female | Jannot, Kerans, *Can. J. Zool.* 81, 1956-1964 (2003). |
| *Neureclipsis crepuscularis* | Invertebrate | length | male, female | Jannot, Kerans, *Can. J. Zool.* 81, 1956-1964 (2003). |
| *Neusticurus ecpleopus* | Reptile | length, mass | male, female | Vitt *et al.*, *Can. J. Zool.* 76, 1671-1680 (1998). |
| *Onicifelis geoggroyi* | Mammal | mass | male, female | Lucherini *et al.*, *Rev. Chil. Hist. Nat.* 79, 169-174 (2006). |
| *Otus asio* | Bird | mass | male, female | Gehlbach, *Southwest Nat.* 48, 70-80 (2003). |
| *Otus Kennicottii* | Bird | mass | male, female | Gehlbach, *Southwest Nat.* 48, 70-80 (2003). |
| *Pan troglodytes schweinfurthii* | Mammal | mass | male, female | Uehara, Nishida, *Am. J. Phys. Anthropol.* 72, 315-321 (1987). |
| *Papio anubis* | Mammal | mass | male, female | Popp, *Primates* 24, 198-210 (1983). |
|  |  |  |  | Eley *et al.*, *Am. J. Primatol.* 18, 209-219 (1989). |
| *Papio cynocephalus* | Mammal | length, mass | male, female | Popp, *Primates* 24, 198-210 (1983). |
|  |  |  |  | Gest, Siegel, *Am. J. Phys. Anthropol.* 61, 189-196 (1983). |
| *Papio hamadryas* | Mammal | mass | male, female | Popp, *Primates* 24, 198-210 (1983). |
| *Papio ursinus* | Mammal | mass | male, female | Popp, *Primates* 24, 198-210 (1983). |
| *Parapediasia teterrella* | Invertebrate | mass | male, female | Marshall, *Am. Midl. Nat.* 119, 412-419 (1988). |
| *Parapsyche elsis* | Invertebrate | length | male, female | Jannot, Kerans, *Can. J. Zool.* 81, 1956-1964 (2003). |
| *Petaurista alborufus* | Mammal | length, mass | male, female | Lee, *Acta Zool. Taiwanica* 9, 51-57 (1998). |
| *Petaurista petaurista* | Mammal | length, mass | male, female | Lee, *Acta Zool. Taiwanica* 9, 51-57 (1998). |
| *Petaurus australis* | Mammal | mass | male, female | Quin *et al.*, *Aust. J. Zool.* 44, 19-45 (1996). |
| *Petaurus breviceps* | Mammal | mass | male, female | Quin *et al.*, *Aust. J. Zool.* 44, 19-45 (1996). |
| *Phalacrocorax atricepts* | Bird | mass | male, female | Rasmussen, *Auk* 111, 143-161 (1994). |
| *Phylocentropus lucidus* | Invertebrate | length | male, female | Jannot, Kerans, *Can. J. Zool.* 81, 1956-1964 (2003). |
| *Phylocentropus placidus* | Invertebrate | length | male, female | Jannot, Kerans, *Can. J. Zool.* 81, 1956-1964 (2003). |
| *Pituophis melanoleucus* | Reptile | length, mass | male, female | Diller, Wallace, *Herpetologica* 52, 343-360 (1996). |
| *Poecilia reticulata* | Fish | length | male, female | McKellar, unpubl. data |
| *Polycentropus cinereus* | Invertebrate | length | male, female | Jannot, Kerans, *Can. J. Zool.* 81, 1956-1964 (2003). |
| *Polycentropus crassicornis* | Invertebrate | length | male, female | Jannot, Kerans, *Can. J. Zool.* 81, 1956-1964 (2003). |
| *Potamyia flava* | Invertebrate | length | male, female | Jannot, Kerans, *Can. J. Zool.* 81, 1956-1964 (2003). |
| *Presbytis obscura* | Mammal | mass | male, female | Burton, *Int. J. Primatol.* 2, 351-368 (1981). |
| *Propithecus diadema edwardsi* | Mammal | length, mass | male, female | Glander *et al.*, *J. Hum. Evol.* 22, 1-17 (1992). |
| *Psacothea hilaris* | Invertebrate | length | male, female | Fukaya *et al.*, *Appl. Entomol. Zool.* 39, 603-609 (2004). |
| *Pseudemys texana* | Reptile | length | male, female | Lindeman, *Southwest Nat.* 52, 586-594 (2007). |
| *Psychomyia flavida* | Invertebrate | length | male, female | Jannot, Kerans, *Can. J. Zool.* 81, 1956-1964 (2003). |
| *Puffinus creatopus* | Bird | length, mass | male, female | Guicking *et al.*, *J. Ornithol.* 145, 64-68 (2004). |
| *Rana limnocharis* | Amphibian | mass | male, female | Wu *et al.*, J. *Anim. Ecol.* 75, 1071-1080 (2006). |
| *Rattus rattus* | Mammal | length, mass | male, female | Ventura, López-Fuster, *Orsis* 15, 91-102 (2000). |
| *Saccostomus campestris* | Mammal | length, mass | male, female | Ellison *et al.*, *Global Ecol. Biogeogr.* 3, 41-47 (1993). |
| *Saguinus oedipus oedipus* | Mammal | mass | male, female | Savage *et al.*, *Am. J. Primatol.* 31, 189-196 (1993). |
| *Saimiri sciureus* | Mammal | length, mass | male, female | Middleton, Rosal, *Lab. Anim. Sci.* 22, 583-586 (1972). |
| *Sceloporus ochoteranae* | Reptile | length | male, female | Smith *et al.*, *Southwest Nat.* 48, 123-126 (2003). |
| *Schistometopum thomense* | Amphibian | length, mass | male, female | Measey, van Dongen, *Evol. Ecol. Res.* 8, 1049-1059 (2006). |
| *Smicridea dispar* | Invertebrate | length | male, female | Jannot, Kerans, *Can. J. Zool.* 81, 1956-1964 (2003). |
| *Smicridea dithyra* | Invertebrate | length | male, female | Jannot, Kerans, *Can. J. Zool.* 81, 1956-1964 (2003). |
| *Smicridea fasciatella* | Invertebrate | length | male, female | Jannot, Kerans, *Can. J. Zool.* 81, 1956-1964 (2003). |
| *Somateria mollissima borealis* | Bird | mass | male, female | Jamieson *et al.*, *Polar Biol.* 29, 585-594 (2005). |
| *Spalax ehrenbergi* | Mammal | length, mass | male, female | Nevo *et al.*, *Z. Zool. Syst. Evol. Forsch.* 26, 286-314 (1988). |
| *Spermophilus columbianus* | Mammal | mass | male, female | Dobson, *Am. Nat.* 140, 109-125 (1992). |
| *Takydromus septentrionalis* | Reptile | length | female | Du *et al.*, *Biol. J. Linnean Soc.* 85, 443-453 (2005). |
| *Tamias alpinus* | Mammal | length | male, female | Levenson, *J. Mammal.* 71, 161-170 (1990). |
| *Tamias canipes* | Mammal | length | male, female | Levenson, *J. Mammal.* 71, 161-170 (1990). |
| *Tamias cinereicollis cinereicollis* | Mammal | length | male, female | Levenson, *J. Mammal.* 71, 161-170 (1990). |
| *Tamias dorsalis* | Mammal | length | male, female | Levenson, *J. Mammal.* 71, 161-170 (1990). |
| *Tamias durangae* | Mammal | length | male, female | Levenson, *J. Mammal.* 71, 161-170 (1990). |
| *Tamias merriami* | Mammal | length | male, female | Levenson, *J. Mammal.* 71, 161-170 (1990). |
| *Tamias minimus* | Mammal | length | male, female | Levenson, *J. Mammal.* 71, 161-170 (1990). |
| *Tamias panamintinus* | Mammal | length | male, female | Levenson, *J. Mammal.* 71, 161-170 (1990). |
| *Tamias ruficaudus* | Mammal | length | male, female | Levenson, *J. Mammal.* 71, 161-170 (1990). |
| *Tamias sibiricus* | Mammal | length | male, female | Levenson, *J. Mammal.* 71, 161-170 (1990). |
| *Tamias speciosus frater* | Mammal | length | male, female | Levenson, *J. Mammal.* 71, 161-170 (1990). |
| *Tamias striatus* | Mammal | length | male, female | Levenson, *J. Mammal.* 71, 161-170 (1990). |
| *Tamias townsendii* | Mammal | length | male, female | Levenson, *J. Mammal.* 71, 161-170 (1990). |
| *Tamias umbrinus* | Mammal | length | male, female | Levenson, *J. Mammal.* 71, 161-170 (1990). |
| *Testudo hermanni* | Reptile | length, mass | male, female | Willemsen, Hailey, *J. Zool. Lond.* 248, 379-396 (1999). |
| *Thamnophis elegans vagrans* | Reptile | length, mass | male, female | Graves, Duvall, *J. Herpetol.* 24, 351-356 (1990). |
| *Thamnophis sirtalis* | Reptile | length, mass | male, female | King, *Herpetologica* 45, 84-88 (1989). |
|  |  |  |  | Krause *et al.*, *J. Zool. (London)* 261, 399-407 (2003). |
| *Thymallus thymallus* | Fish | length, mass | female | Haugen, Vollestad, *J. Evol. Biol.* 13, 897-905 (2000). |
| *Trachemys scripta* | Reptile | length | female | Tucker *et al.*, *J. Herpetol.* 32, 294-298 (1998). |
| *Troglodytes aedon* | Bird | mass | male, female | Tieleman *et al.*, *Funct. Ecol.* 20, 491-499 (2006). |
| *Ursus arctos* | Mammal | length | female | Zedrosser *et al.*, *J. Mammal.* 87, 510-518 (2006). |
| *Uta antiqua* | Reptile | length | male, female | Dunham *et al.*, *Ecology* 59, 1230-1238 (1978). |
| *Uta molascensis* | Reptile | length | male, female | Dunham *et al.*, *Ecology* 59, 1230-1238 (1978). |
| *Uta palmeri* | Reptile | length | male, female | Dunham *et al.*, *Ecology* 59, 1230-1238 (1978). |
| *Uta squamata* | Reptile | length | male, female | Dunham *et al.*, *Ecology* 59, 1230-1238 (1978). |
| *Uta stansburiana* | Reptile | length | male, female | Dunham *et al.*, *Ecology* 59, 1230-1238 (1978). |
| *Vulpes vulpes* | Mammal | length, mass | male, female | Gortazar *et al.*, *J. Zool. (London)* 250, 335-338 (2000). |
