# Supplementary material for: How Humans Differ from Other Animals in Their Levels of Morphological Variation
Source: PLoS One. 2009 Sep 1;4(9):e6876. doi: 10.1371/journal.pone.0006876 (PMC2730817; doi:10.1371/journal.pone.0006876)
Supplement: Table S2 — List of all studies used to obtain CVs for male and/or female height and/or mass for human populations. Also included is the country of origin, name of specific population or survey title, year of sampling (if provided), indigenous/aboriginal status (as defined in each study), and development status (http://www.un.org/special-rep/ohrlls/ldc/list.htm). (0.11 MB DOC) [file pone.0006876.s002.doc]

**Table S2.** **List of all studies used to obtain CVs for male and/or female height and/or mass for human populations**. Also included is the country of origin, name of specific population or survey title, year of sampling (if provided), indigenous/aboriginal status (as defined in each study), and development status (http://www.un.org/special-rep/ohrlls/ldc/list.htm).

| Country (Specific population or survey title, year of sampling if provided) | Indigenous/aboriginal | Least Developed Country | Trait | Sex | Reference |
| --- | --- | --- | --- | --- | --- |
| Australia (Njalia) | Y | N | height, mass | male, female | Abbie, *Oceania* 27, 220-243 (1957). |
| Benin (Manta, 1990-1991) | Y | Y | height, mass | female | Ategbo *et al.*, *Am. J. Clin. Nutr.* 61, 466-472 (1995). |
| Iran (Shiraz, 1988-1989) | N | N | height, mass | male, female | Ayatollahi, Carpenter, *Ann. Hum. Biol.* 20, 13-19 (1993). |
| Yemen (Aden, Dar Sa’ad, Rural, 1982-1983) | N, N, N | Y, Y, Y | height, mass | female | Bagenholm *et al.*, *Bull. World Health Organ.* 66, 491-498 (1998). |
| Tanzania (Hadza, 1966-1967); Uganda (Baganda,1966-1967) | N, Y | Y, Y | height, mass | male | Barnicot *et al.*, *Hum. Biol.* 44, 87-116 (1972). |
| China (China Health and Nutrition Survey, 1997); United States (Third National Health and Nutrition Examination Survey, 1988-1994); Philippines (Cebu Longitudinal Health and Nutrition Survey, 1998) | N, N, N | N, N, N | height, mass | male, female | Bell *et al.*, *Am. J. Epidemiol.* 155, 346-353 (2002). |
| Canada (Ahousat, Anaham) | N | N | height, mass | male, female | Birkbeck *et al.*, *Can. J. Public Health* 62, 403-414 (1971). |
| Central African Republic (Western Pygmies, C.A.R. farmers, 1969); Democratic Republic of the Congo (Eastern Pygmies, 1971) | Y, Y, Y | Y, Y, Y | height, mass | male, female | Cavalli-Sforza, *African Pygmies*, p.81-93 (1986). |
| India (Calcutta) | N | N | height, mass | female | Chatterjee, Saha, *Ann. Hum. Biol.* 20, 31-38 (1993). |
| China (Taiwan, 1954) | N | N | height, mass | male, female | Chen *et al.*, *Ann. N.Y. Acad. Sci.* 110, 760-777 (1963). |
| Fiji (Suva, 1985) | Y | N | height, mass | male, female | Clegg, *Ann. Hum. Biol.* 16, 507-528 (1989). |
| Mali (Torokoro, Merediela, N’tenkoni, Famabougou, Dogo, Siramana, 1989) | N, N, N, N, N, N | Y, Y, Y, Y, Y, Y | height, mass | male, female | Dettwyler, *Am. J. Phys. Anthropol.* 88, 309-321 (1992). |
| Guatemala (Western Highlands) | N | N | height, mass | male, female | Diaz *et al.*, *Am. J. Hum. Biol.* 3, 525-530 (1991). |
| Democratic Republic of the Congo (Efe pygmies, Lese, 1980) | Y, Y | Y, Y | height, mass | male, female | Dietz *et al.*, *Am. J. Phys. Anthropol.* 78, 509-518 (1989). |
| Colombia (Cali, 1988-1989) | N | N | height, mass | female | Dufour *et al.*, *Am. J. Hum. Biol.* 6, 749-760 (1994). |
| Peru (Nuñoa, 1964-1966) | Y | N | height, mass | male | Frisancho, Baker, *Am. J. Phys. Anthropol.* 32, 279-292 (1970). |
| Burkina Faso (Bella, Rimaibe, Malebe, Sonrai, Ful, Mossi of Donse, Gurmanche, Bwaba, Mossi of Kokologo); Mali (Dogon) | N, N, N, N, N, N, N, N, N, N | Y, Y, Y, Y, Y, Y, Y, Y, Y, Y | height, mass | male, female | Froment, Hiernaux, *Ann. Hum. Biol.* 11, 189-200 (1984). |
| Nepal (Upper Khumbu Sherpas); India (Kalimpong Sherpas) | Y, Y | Y, Y | height, mass | male, female | Gupta, Basu, *Ann. Hum. Biol.* 8, 145-151 (1981). |
| Ethiopia (Adi-Arkai, Debarech, 1965-1966) | N, N | Y, Y | height, mass | male, female | Harrison *et al.*, *Philos. Trans. R. Soc. Lond. B* 256, 147-182 (1969). |
| Venezuela (Carabali, San Esteban, 1985) | N, N | N, N | height, mass | male, female | Hurtado, Hill, *Hum. Ecol.* 15, 163-187 (1987). |
| India (Gujarat) | N | N | height, mass | male, female | Kaur, Singh, *Ann. Hum. Biol.* 8, 333-339 (1981). |
| Canada (Upper Liard, Ross River, Ft. St. John) | Y, Y, Y | N, N, N | height, mass | male, female | Lee, Birkbeck, *Hum. Biol.* 49, 581-591 (1977). |
| Russia (Evenki, 1991-1992) | Y | N | height, mass | male, female | Leonard *et al.*, *Am. J. Hum. Biol.* 6, 339-350 (1994). |
| United States (University students); Kenya (Turkana) | N, N | N, N | height, mass | male, female | Little, Johnson *Am. J. Phys. Anthropol.* 69, 335-344 (1986). |
| South Africa (Venda) | N | N | height, mass | male | Loots, Lamprecht, *S. Afr. Med. J.* 45, 1284-1288 (1971). |
| Israel (Kurdish Jews, Yemenite Jews, 1968) | N, N | N, N | height, mass | male, female | Lourie, *Philos. Trans. R. Soc. Lond. B* 266, 101-112 (1973). |
| Papua New Guinea (Wopkaimin, 1982-1983) | Y | N | height, mass | male, female | Lourie *et al.*, *Ann. Hum. Biol.* 13, 517-536 (1986). |
| Papua New Guinea (Kaiapit, Bundi, Kukukuku, 1965-1967) | N, N, N | N, N, N | height, mass | male, female | Malcolm, *Am. J. Phys. Anthropol.* 31, 39-52 (1969). |
| Papua New Guinea (Asai valley) | N | N | height, mass | male, female | Malcolm, *J. Biosoc. Sci.* 2, 213-226 (1970). |
| Mexico (Valley of Oaxaca, 1971-1972, 1978) | N | N | height | male, female | Malina, *et al.*, *Am. J. Phys. Anthropol.* 60, 437-449 (1983). |
| Nepal (Kathmandu, 1984-1985) | N | Y | height, mass | female | Malville*, Am. J. Hum. Biol*. 3, 377-387 (1991). |
| United States (Tutuila, 1990) | N | N | height, mass | male, female | McGarvey *et al.*, *Am. J. Hum. Biol.* 5, 17-30 (1993). |
| Brazil (Tukanoan, Maku, 1981) | Y, Y | N, N | height, mass | male, female | Milton, *Ann. Hum. Biol.* 10, 435-440 (1983). |
| Japan (Air Force) | N | N | height, mass | male | Miyashita, Takahashi, *Hum. Biol.* 43, 327-339 (1971). |
| United Kingdom (British Petroleum Company Limited, 1964, 1966) | N | N | height, mass | male, female | Montegriffo, *Ann. Hum. Genet.* 31, 389-399 (1967). |
| Chile (Aymara, 1972) | Y | N | height, mass | male, female | Mueller *et al.*, *Ann. Hum. Biol.* 5, 329-352 (1978). |
| Bolivia (Bolivian Altiplano, 1975) | N | N | height, mass | male, female | Mueller *et al.*, *Hum. Biol.* 52, 529-546 (1980). |
| The Netherlands (Dutch nationwide growth study, 1997) | N | N | height | male, female | Niewenweg *et al.*, *Ann. Hum. Biol.* 30, 563-569 (2003). |
| Brazil (Simões Lopes) | Y | N | height, mass | male, female | Niswander *et al.*, *Am. J. Hum. Genet.* 19, 490-501 (1967). |
| Papua New Guinea (Kaul, Lufa) | N, N | N, N | height, mass | male, female | Norgan *et al.*, *Philos. Trans. R. Soc. Lond. B* 268, 309-348 (1974). |
| Samoa (Salamumu); United States (Manu’a, Hawaii, California) | N, N, N, N | Y, Y, N, N | height, mass | male, female | Pawson, *The Changing Samoans: Behaviour and Health in Transition*, p. 254-274 (1986). |
| Samoa (Neiafutai, 1986-1987); United States (Honolulu, 1986-1987) | N, N | Y, N | height, mass | male, female | Pearson, *Am. J. Hum. Biol.* 2, 313-326 (1990). |
| Malawi (Malawi Maternal and Child Nutrition Study, 1986-1987) | N | Y | height, mass | male, female | Pelletier *et al.*, *Am. J. Hum. Biol.* 3, 347-361 |
| Australia (Rembarranga, 1969); Czech Republic (State-wide survey, 1961 and 1971) | Y, N | N, N | height, mass | male, female | Prokopec, *J. Hum. Evol.* 6, 371-391 (1977). |
| Sudan (Shilluk, Ruweng Dinka, Ageir Dinka, 1953-1954) | Y, Y, Y | Y, Y, Y | height, mass | male | Roberts, Bainbridge, *Am. J. Phys. Anthropol.* 21, 341-370 (1963). |
| Canada (Igloolik Inuit, Volochanka nGanasan, 1992) | N, N | N, N | height, mass | male, female | Rode, Shephard*, Int. J. Obes.* 19, 709-716 (1995). |
| Uganda and Kenya (Osteological collections from Makerere University, 1945-1995) | N | N | height, mass | male, female | Ruff, *J. Hum. Evol.* 38, 269-290 (2000). |
| India (Dehli) | N | N | height, mass | female | Singh, Raja, 1980 *Eur. J. Appl. Physiol.* 43, 69-81 (1980). |
| India (Madras, Ooty, 1972-1973) | N | N | height, mass | male | Singh, *Ann. Hum. Biol.* 2, 301-304 (1975). |
| Chile (Mapuche) | N | N | height | male, female | Soto-Heim, *Biom. Hum. Anthropol.* 20, 235-246 (2002). |
| Canada (Dogrib Indians, 1979) | Y | N | height, mass | male, female | Szathmary, Holt, *Hum. Biol.* 55, 493-515 (1983). |
| Pakistan (Lahore, 1962-1964) | N | N | height, mass | male, female | Underwood *et al.*, *Am. J. Clin. Nutr.* 20, 694-701 (1967). |
| Kenya (Ngayu pygmies, Bayenga Pygmies, Bantu, 1956-1959) | Y, Y, Y | N, N, N | height, mass | female | Vincent, *Am. J. Phys. Anthropol.* 20, 237-247 (1962). |
| Namibia (¡Kung San, 1987) | N | N | height, mass | male | Winkler, Kirchengast, *Am. J. Hum. Biol.* 6, 203-213 (1994). |
| Chile (Ollagüe); Nepal (Khunde) | N, N | N, Y | height, mass | male | Winslow *et al.*, *Am. J. Hum. Biol.* 2, 653-662 (1990). |
