# Supplementary material for: How Humans Differ from Other Animals in Their Levels of Morphological Variation
Source: PLoS One. 2009 Sep 1;4(9):e6876. doi: 10.1371/journal.pone.0006876 (PMC2730817; doi:10.1371/journal.pone.0006876)
Supplement: Table S3 — Percentiles for mean within- and among-population male and female human height and mass in relation to species-mean amphibian, invertebrate, mammal, and reptile length and mass distributions. Percentiles are not shown for taxa distributions with n<5 animal species. (0.03 MB DOC) [file pone.0006876.s003.doc]

**Table S3. Percentiles for mean within- and among-population male and female human height and mass in relation to species-mean amphibian, invertebrate, mammal, and reptile length and mass distributions**. Percentiles are not shown for taxa distributions with n < 5 animal species.

|  |  | Amphibian | Invert | Mammal | Reptile |
| --- | --- | --- | --- | --- | --- |
| Within populations | Male height | 0^th^ | 0^th^ | 18^th^ | 0^th^ |
|  | Female height | 0^th^ | 0^th^ | 5.5^th^ | 5.7^th^ |
|  | Male mass | - | - | 59^th^ | 0^th^ |
|  | Female mass | - | 45^th^ | 66^th^ | 0^th^ |
| Among populations | Male height | 19^th^ | 39^th^ | 61^st^ | 21^st^ |
|  | Female height | 9.5^th^ | 47^th^ | 57^th^ | 41^st^ |
|  | Male mass | - | - | 54^th^ | 31^st^ |
|  | Female mass | - | - | 79^th^ | 65^th^ |
