# Supplementary figures and images for: How Humans Differ from Other Animals in Their Levels of Morphological Variation
Source: PLoS One. 2009 Sep 1;4(9):e6876. doi: 10.1371/journal.pone.0006876 (PMC2730817; doi:10.1371/journal.pone.0006876)

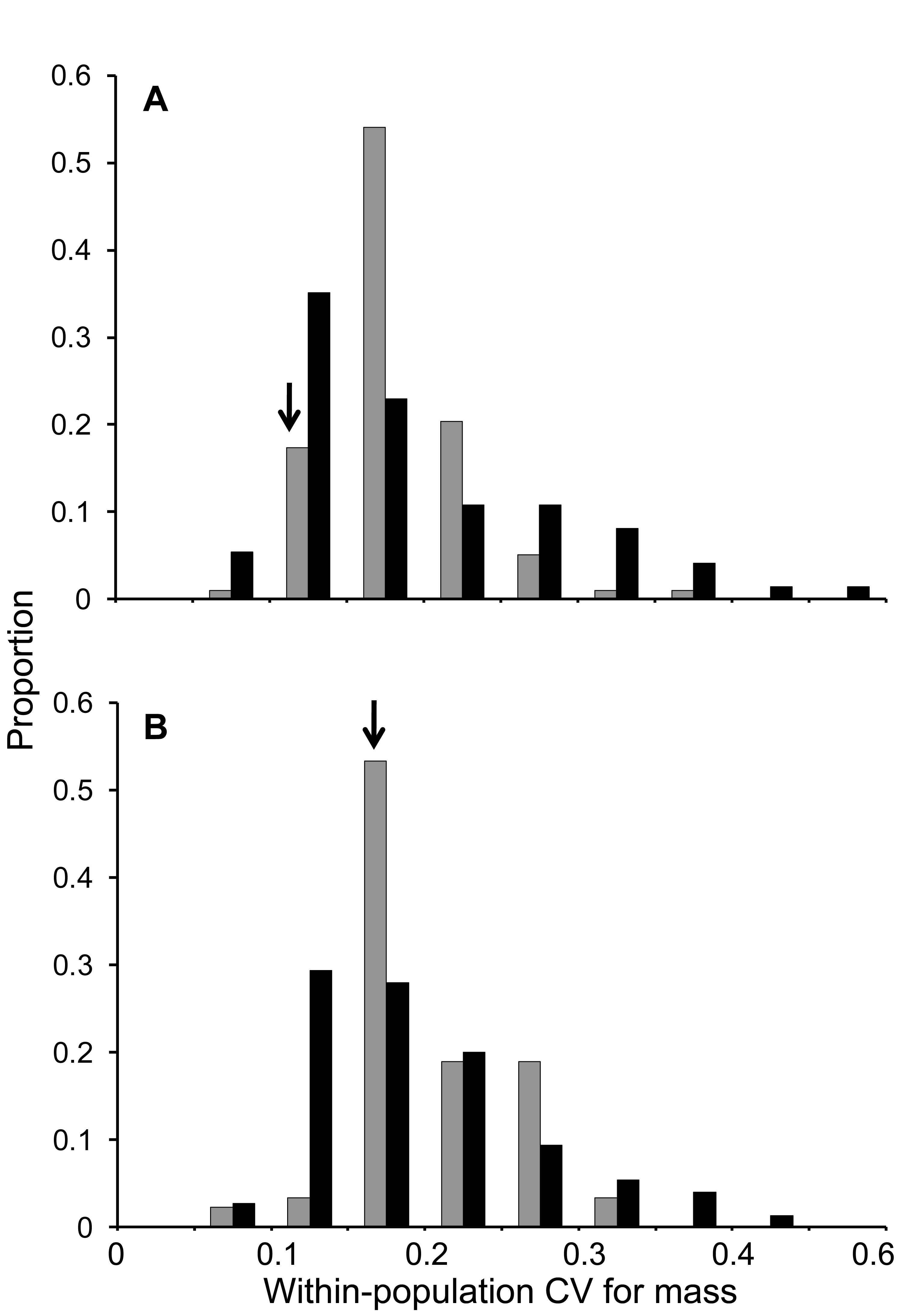

Supplement: Figure S1 — Distributions of coefficients of variation (CV) for within-population body mass. Shown are species means for animals (black) and population means for humans (grey) for males (A) and females (B). Arrows indicate the locations of CVs for mean human mass. (1.80 MB TIF) [file pone.0006876.s004.tif]

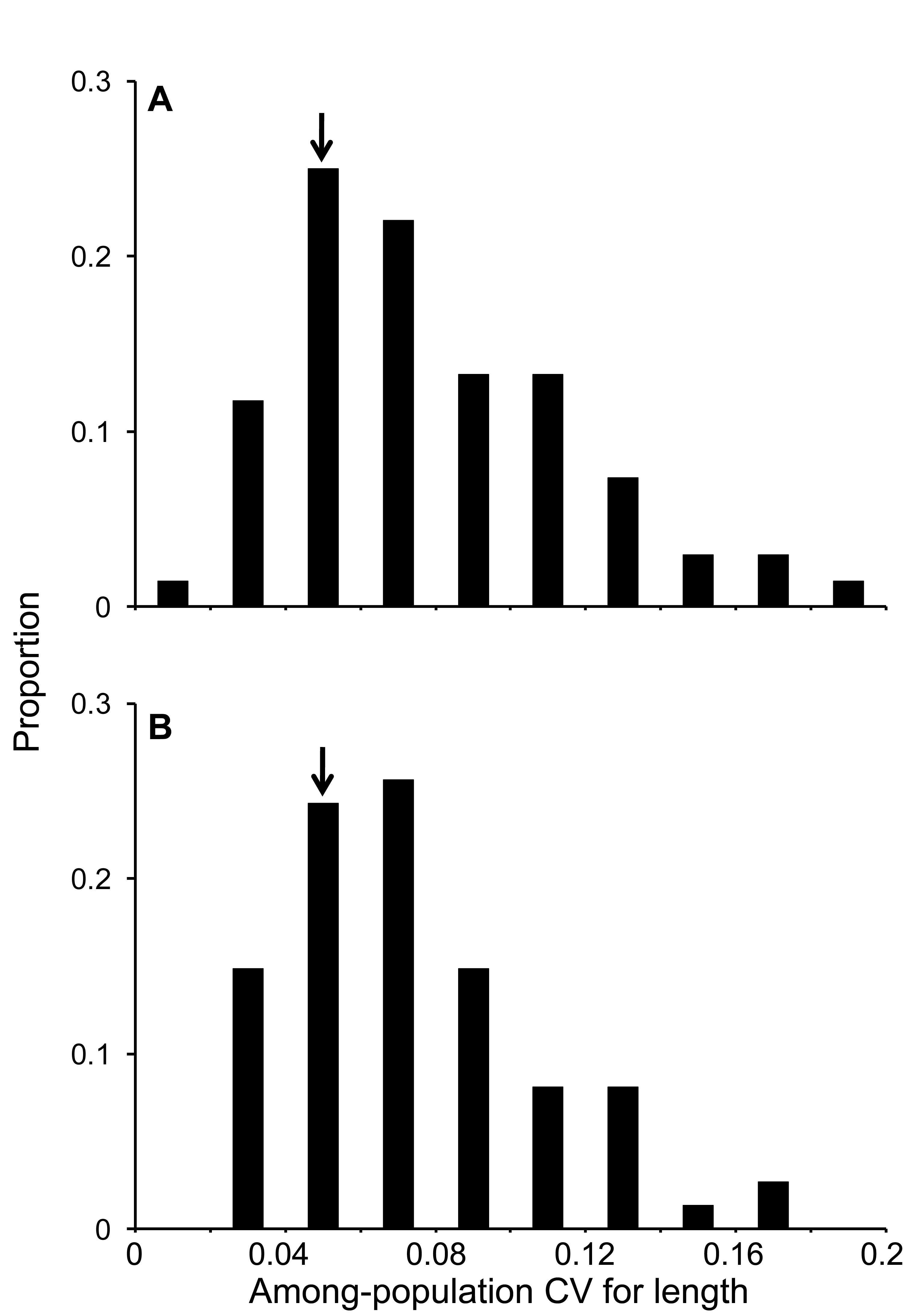

Supplement: Figure S2 — Distributions of CVs for among-population body length or height. Shown are data for males (A) and females (B). Arrows indicate the locations of CVs for mean human height. (1.72 MB TIF) [file pone.0006876.s005.tif]

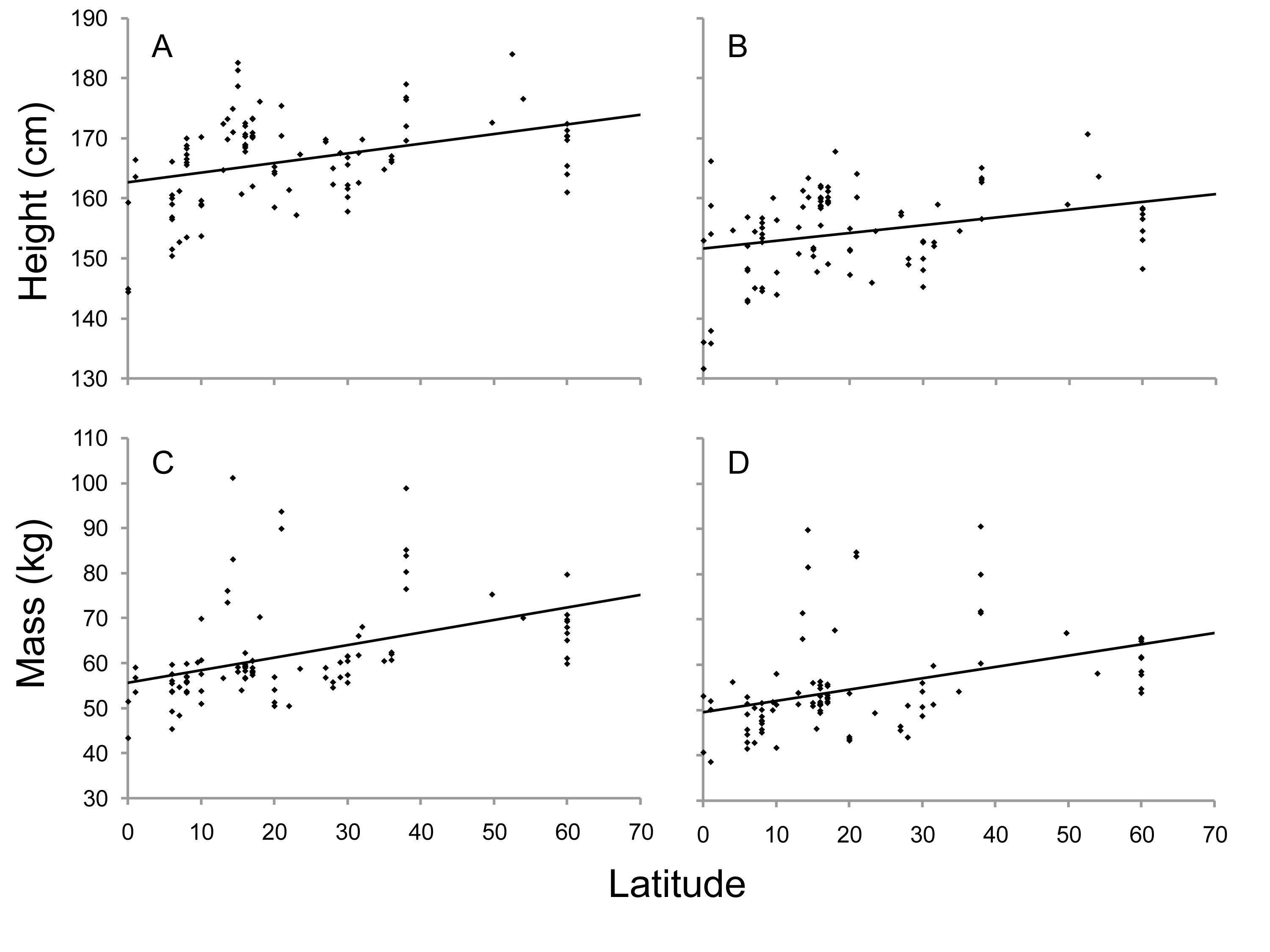

Supplement: Figure S3 — Bergmann's rule in humans. Mean male height (A, R2 = 0.126, P<0.001), female height (B, R2 = 0.097, P = 0.002), male mass (C, R2 = 0.183, P<0.001), and female mass (D, R2 = 0.155, P<0.001) all increase significantly with absolute latitude. Latitude of each population was approximated using the geographic centre of the country from which the population was sampled. Coordinates were obtained from the CIA World Factbook (https://www.cia.gov/library/publications/the-world-factbook/fields/2011.html). (1.06 MB TIF) [file pone.0006876.s006.tif]

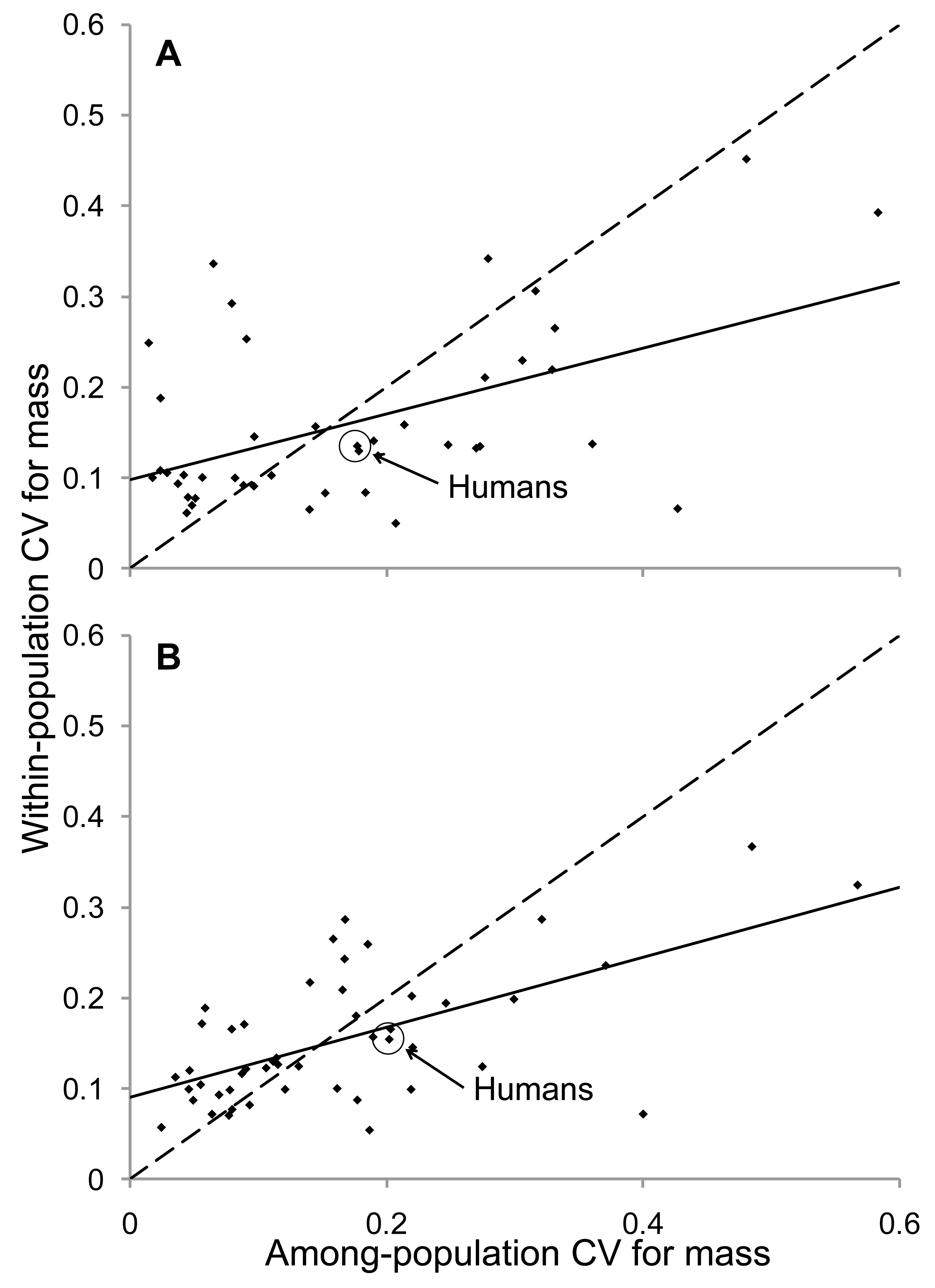

Supplement: Figure S4 — Species-mean CVs for among- versus within-population body mass. Shown are regression lines (solid), x = y lines (dashed), and data for males (A, R2 = 0.26, P<0.001) and females (B, R2 = 0.37, P<0.001). (1.72 MB DOC) [file pone.0006876.s007.tif]
